# Natural Dyeing of Cellulose and Protein Fibers with the Flower Extract of *Spartium junceum* L. Plant

**DOI:** 10.3390/ma14154091

**Published:** 2021-07-22

**Authors:** Zorana Kovačević, Ana Sutlović, Ana Matin, Sandra Bischof

**Affiliations:** 1 Department of Textile Chemistry and Ecology, Faculty of Textile Technology, University of Zagreb, 10000 Zagreb, Croatia; zorana.kovacevic@ttf.unizg.hr (Z.K.); ana.sutlovic@ttf.unizg.hr (A.S.); 2 Department of Agricultural Technology, Storage and Transport, Faculty of Agriculture, University of Zagreb, 10000 Zagreb, Croatia; amatin@agr.hr

**Keywords:** *Spartium junceum* L., natural dye, mordant dye, cellulose fibers, protein fibers, wool dyeing, flavonoids, colorfastness to washing

## Abstract

In this study, the natural dye was extracted from *Spartium junceum* L. (SJL) flowers and applied on cellulose (cotton) and protein (wool) fabric. Fabrics were pre-mordant with alum prior to the dyeing process. Considering the global requirements on zero waste and green policy, the dyeing process was intended to be as much as possible environmentally friendly but still effective. Therefore, mordant concentration was optimized due to the reduction of the negative impact. The efficiency of the dyeing process was investigated by examination of fabrics’ color characteristics and colorfastness to washing properties. In this paper, we have proved that the extracted dye from *Spartium junceum* L. is an acidic dye (mordant dye) which is more applicable for the treatment of wool fabrics. In this paper, it was proved that phytochemicals responsible for coloring are part of the flavonoids group. The UV absorption spectra of extracted dye show 4 bands in the region of λ_max_ 224, 268, 308 and 346 nm which are ascribed to bands characteristic for flavonoids. Wool fabric pre-mordant with 3% alum and dyed shows great chromatic (C*) properties where C* value is in a range from 47.76 for unwashed samples to 47.50 for samples after 5 washing cycles and color hue (h°) is in a range 82.13 for unwashed samples to 81.52 for samples after 5 washing cycles. The best result regarding the colorfastness properties is shown by the wool sample treated with 3% alum after 5 washing cycles (total difference in color (Delta E*) = 0.87). These results confirm that metal (Al) from alum mordant make strong chemical bonds with wool substrate and dye since Delta E* values decrease in comparison to Delta E* values of the cotton samples treated the same way. The results revealed it is possible to reduce the concentration of mordant up to 3% and obtain satisfactory results regarding the colorfastness. Nevertheless, future research will go in the direction of replacing synthetic mordant with a more environmentally friendly one.

## 1. Introduction

Since an interdisciplinary approach is desirable within the science community, this research comprises several specific objectives, which seeks to find new solutions for a better understanding of the investigated topic. The wide availability of indigenous Mediterranean plants directs research towards sustainable development and circular economy. Furthermore, utilization of widely available biomass for natural dye production will be applied in the dyeing of protein and cellulosic textile materials with emphasis on the aspects of textile care.

*Spartium junceum* L. (SJL) or Spanish broom is a Mediterranean plant, which has been used as a textile raw material since ancient times. Its greatest use was in fiber production but it was also well known for its flowers which are blooming between May and July [1]. It is interesting that the SJL plant as a raw material resource was mostly used in the period of poverty between the great wars but afterward was abandoned. Although, it is native to southern Europe and the Mediterranean area, including North Africa, Turkey and the Middle East it becomes an aggressive invader in many tropical, subtropical and temperate regions of the world. For example, in the USA, the Canary Islands, the Azores, Argentina, Bolivia, Peru, Uruguay, South Africa and the Dominican Republic SJL is considered an invasive, noxious weed [2,3]. Nowadays, SJL is applied again as a renewable raw material which could have numerous possibilities of usage for the production of fibers, yarns, fabrics, ropes, baskets, composite materials reinforced with SJL fibers [1], decorative and artistic items, painting canvas, oils, perfumes, and natural dyes by following the concept of circular economy [4] and zero waste model. Aqueous extracts prepared from the flowers were used as herbal medicine in the treatment of gastric ulcers in Turkish folk medicine, against herpes simplex virus type 1 in recent times [5] and in yellow dye production [6,7]. Regardless of the fact that natural dyes were successfully used for fabric dyeing in ancient times, the discovery of synthetic dyes in the 19th century significantly improved and facilitated the dyeing process by achieving cheaper dyes. Due to increased awareness of the environmental and health hazards and cognition of toxicity and carcinogenicity of synthetic dyes products, most commercial dyers have started to search up again for renewable and sustainable natural dyes derived from the plant, animal and mineral sources in accordance with EU regulations. REACH (EC No. 1907/2006) and Ecolabel (EC No. 66/2010) regulations provide restrictions on the usage of dyes that are considered dangerous to human health [8,9]. EU Directives must be implemented by the EU member states in their national legislation and monitored by the national authorities. 

Natural dyes show two main advantages compared to synthetic dyes: synthesis processes within natural materials performed by nature without environmental pollution and biodegradability of natural materials, thus not affecting hazardous effluent upon degradation in the environment [10]. The limitation of natural dyes upon the synthetic one is their reproduction of color since the quality of natural dyes depends on the climate, plant genus, region, etc. [10,11]. The dyeing of textile materials is influenced by dyeing parameters such as fiber structure, temperature, time and pH of the dye bath and dye molecule characteristics. The efficiency of dyes on textile materials depends on the stability of the dyes/fiber complex. Cellulose fibers show poor affinity and substantivity according to natural dyes causing difficulties in the dyeing process. Protein fibers, on the contrary, show better affinity to natural dyes since they have ionic groups in their structure and get stronger bonds with natural dyes possessing ionic groups in dye structure [12,13].

Natural dyes can be classified based on their chemical structure, origin, method of application and color. Based on the chemical structure they belong to classes of indigoids, anthraquinonoids, ketones, imines, betalains, anthocyanidins, flavonoids, carotenoids or chlorophylls. Based on their method of application they can be classified as mordant dyes, direct dyes, vat dyes, acid dyes, basic dyes and disperse dyes [14,15]. According to the literature [16] flavonoids (water soluble polyphenols) give yellow color to SJL flowers. Chemically flavonoids are divided into flavones, flavonols, isoflavones, flavanones, chalcones and arones. The SJL plant’s dried flowers contain luteolin and quercetin dyes. Luteolin is part of the flavones subclass, which are UV absorbing flavonoids, while quercetin is part of the flavonols subclass. Today, in addition to aqueous extraction, modern methods are used for the extraction of pigments from plant sources, e.g., microwaves [17], ultrasound [18], supercritical CO_2_ [19]. These methods are used for qualitative analysis, however, in the application of these components in the field of microbiology and green synthesis, aqueous extraction is still preferred. Quercetin and luteolin belong to the mordant dyes class [20], which is equally suitable for both vegetable and animal fibers [21]. In most ways, mordant dyes show soft, pastel and lustrous shades soothing to the human eye but one of their main drawbacks is little affinity for textile substrates causing poor colorfastness to washing, rubbing, sweat and sunlight [22]. Mordant dyes are acid dyes and have an anionic character, therefore requiring mordants in their formulations to fix dyes on fibers and improve the quality and fabric’s color brightness [20,21,22,23]. Mordants can be natural (tannic acid, citric acid, clay, bark extracts, etc.) or synthetic (various metallic salts, such as alum, stannous chloride, copper sulfate, ferrous sulfate, etc.) [23]. Metallic mordants are usually used for fabrics pretreatment before the dyeing process. Different metal mordants impact various coloration effects with the same natural dye. The final color experience does not depend only on a dye but is also affected by the type and concentration of mordant. In this paper, we have used alum, metallic salt of potassium aluminum sulfate dodecahydrate (KAl(SO_4_)_2_ x12 H_2_O). It is a widely used mordant for cotton and wool fibers which show the lowest negative ecological impact among other metallic mordants [24]. It is recommended to add approx. 12% of alum on the weight of the fabric [25] but in this paper, we have investigated the color enhancement of pre-mordant fabrics with a much lower amount of alum (3 to 5%) and color changes of such fabrics after the washing process. Studies about SJL dye’s colorfastness to washing are very rare in the literature. In this paper, the possibility of the usage of the aqueous extract from the SJL flowers as a natural dye for the cellulose and protein fabrics was evaluated. The novelty of this work is in the direct contribution towards the principles of a circular economy where each part of the plant is successfully utilized for the production of fibers and/or technical textiles, so as for the extraction of natural dyes.

## 2. Experimental

### 2.1. Materials

Two different textile fabrics were used in the experiment: cotton and wool fabrics with characteristic parameters presented in Table 1.

**Figure 1:**
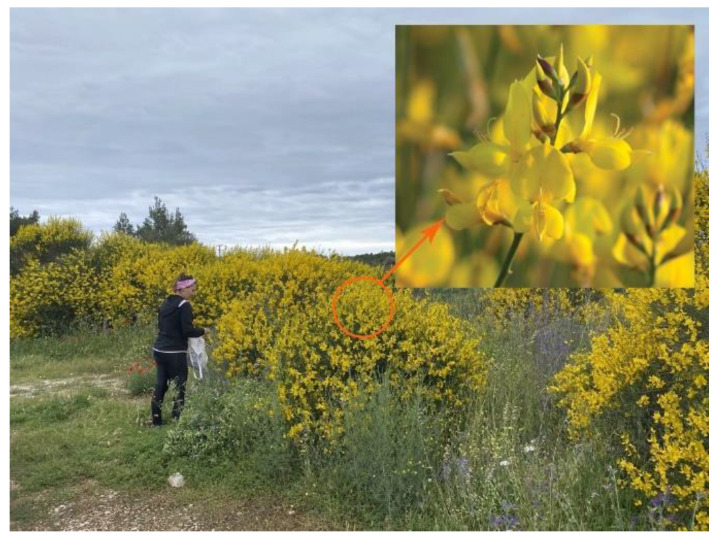
Collecting of *Spartium junceum* L. flowers in May 2021.

*Spartium junceum* L. flowers were picked near the town of Šibenik (Figure 1).

### 2.2. Natural Dye Extraction

*Spartium junceum* L. flowers were used as an herbal source for biological pigment extraction. The dye was extracted by boiling flowers in distilled water at material-liquor ratio (MLR) 1:2.7, at 100 °C for 1 h. After the first extraction process, the dye solution was filtered and evaporated water was adjusted with distilled water to a volume of 3 L. Extraction process was repeated twice by using residual flowers after the first extraction.

#### 2.2.1. Determination of pH Values

The pH values of dye solutions were measured by pH meter MA 5736, Metrel (Iskra), Slovenia with the glass-electrode method. Prior to the measurement pH meter was calibrated in the solution of pH 3, pH 5 and pH 7 buffers.

#### 2.2.2. Determination of the Crude Dye Yield

The mass of the crude dye was determined by the evaporation method. To do this, 100 mL of the extracted and filtered dye solution was placed in the Petri dish and heated in the oven at 105 ± 2 °C for 24 h. Crude solid dry material weight after evaporation was calculated on the volume of 3 L. The solid and dried dye was weighed, and the percentage of crude dye was calculated using Equation (1) in relation to the original weight of plant material used for extraction.

Wcd = (Wae)/Wbe) ∗ 100,(1)

where Wcd–percentage yield of crude dye (%); Wae–crude solid dry material weight after evaporation (g); Wbe–dry material weight before extraction (g). 

#### 2.2.3. FT-IR Spectroscopy

Fourier transform infrared (FT-IR) spectra were obtained with a Perkin Elmer Spectrum 100 FT-IR spectrometer using ATR (attenuated total reflection) method. The analyses were carried out at room temperature and ambient humidity. All spectra were registered from 4000 cm^−1^ to 380 cm^−1^, with a resolution of 4 cm^−1^. The background was collected at the beginning of the measurement. Each spectrum was collected from an average of 4 scans.

#### 2.2.4. UV-VIS Spectrophotometry

The absorption spectra of the dye solutions were measured using a UV-Vis spectrophotometer, UV-2600, Shimadzu Europe, Germany at a wavelength from 200 to 800 nm.

### 2.3. Fabric Treatment

#### 2.3.1. Pre-Mordanting Process

Pre-mordanting of cotton and wool fabrics was applied by using potassium aluminum sulfate dodecahydrate KAl(SO_4_)_2_ × 12 H_2_O. MLR ratio was 1:40, at 50 °C for 30 min with mordant concentrations of 3% and 5% on the weight of the fabric. After mordanting, fabrics were air dried.

#### 2.3.2. Dyeing Process

Cotton and wool fabrics were dyed in water extract of *Spartium junceum* L. flowers by exhaustion method with MLR 1:40, at 90 °C for 60 min. Dyeing was performed with and without mordanting process using dye solution after the first extraction. Afterward, the fabrics were thoroughly washed in cold water, followed by hot soaping with Kemopon 30, CHT Bezema, Switzerland and cold rinsing.

#### 2.3.3. Washing Process

The washing process was carried out at MLR 1:20, at 60 °C for 30 min, through 5 cycles using the apparatus Turbomat P4502, Mathis, Switzerland. The washing bath was made with 2.5 g/L ECE-2 colorfastness test detergent, without using phosphate, ISO 105-C08/C09. Washed fabrics were dried between the washing cycles. Colorfastness to washing was tested by colorimetric evaluations.

#### 2.3.4. Color Measurement

The color coordinates of dyes were determined on spectrophotometer Datacolor 850, Switzerland under illuminant D65, using d/10° geometry. The coordinates used to determine color values are “L*” for lightness, “a*” for redness (positive value) and greenness (negative value), “b*” for yellowness (positive value) and blueness (negative value), “C*” for chroma and “h°” for hue angle in the range of 0° to 360°.

The changes in coloration of unwashed and washed samples were characterized by the difference in lightness “dL*”, hue “dH*”, chroma “dC*” and the total difference in color “Delta E*”. 

Delta E (∆E*) was calculated by the following Equation (2):

∆E* = [(∆L*)^2^ + (∆a*)^2^ + (∆b*)^2^]^1/2^, (2)

where ∆L* = L* washed–L* unwashed; ∆a* = a* washed–a* unwashed; ∆b* = b* washed–b* unwashed.

Color strength (K/S) was calculated by the Kubelka-Munk Equation (3):

K/S = (1 − R)^2^/2R, (3)

where K is the absorption coefficient, S is the scattering coefficient and R is the remission value (reflectance of the dyed fabric) at ʎ_max_. 

The color coordinates were measured before and after the 1st, 3rd and 5th washing cycle.

## 3. Results and Discussion

### 3.1. Sample Labeling

Samples labels were defined according to material composition, dye extraction, pre-mordanting and washing process:

C/W_E_Me_Wx,

where C/W stands for fiber type (C for cotton and W for wool fabric), E stands for the extraction process of dye solutions (1E for 1st extraction, 2E for 2nd extraction and 3E for 3rd extraction), Me is mordant concentration (from 0 to 5% on the weight of the fabric) and Wx is number of washing cycles (from 0 to 5 cycles).

### 3.2. Analysis of Dye Extract

#### 3.2.1. pH Values of Aqueous Dye Solutions

All the investigated solutions show pH values around 5 indicating the acidic range. 1st extraction (1E) shows pH 5.4 at 21.3 °C, 2nd extraction (2E) shows pH 5.5 at 20.6 °C and 3rd extraction (3E) shows pH 5.6 at 22.9 °C. Acidic dye solutions are most often anionic in nature and are suitable for the dyeing of protein fibers and polyamide as well [26,27].

#### 3.2.2. Crude Dye Yield

**Figure 2:**
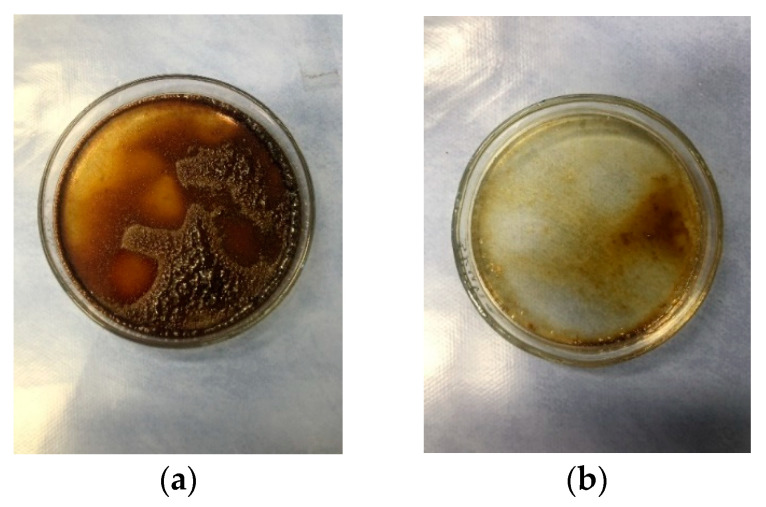
Evaporated residue of (**a**) 1st extraction dye solution (1E) and (**b**) 3rd extraction dye solution (3E).

Crude dye yield is an indicator of the total extracted pigments from the plant source. It significantly depends on the characteristics of the plant (climate, harvesting time, etc.) and on the extraction process parameters (pH, temperature, time) as well. The highest amount of extracted pigment which can be objectively assessed from the Table 2 and visually from Figure 2 is obtained after 1st extraction.

Natural dyes of plant origin, most commonly from the flavonoid group, have been shown to have good solubility in acidic media since glycosides are resistant to acid hydrolysis resulting in better pigment extraction [6,12,16,28,29]. Alkaline media is used in the extraction of compounds, which contain phenolic groups [29,30]. Therefore, flavonoid derivatives are most often detected in the acidic extract. In this paper, a relatively small amount of crude dye yield was obtained after 1st acidic extraction (4.8%), which is further reduced by 50% in each subsequent dyeing process. The results after crude dye yield calculation indicate the unprofitability of using 2E and 3E dye solution, therefore multiple extractions of the collected plant are not recommended for further research. As can be seen from Figure 2, 3E dye solution is inadequate so 1E dye solution was applied in this experiment. This was also confirmed by VIS spectrophotometric analysis of the extracts.

#### 3.2.3. UV-VIS Spectrophotometry

**Figure 3:**
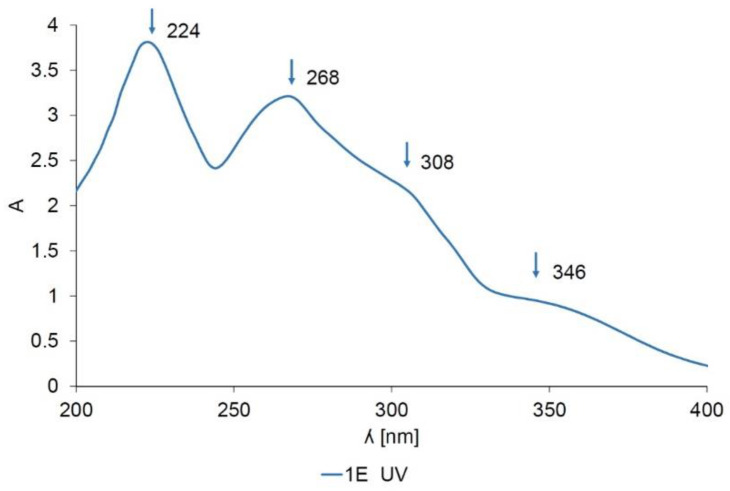
The UV absorption spectra of dye solution 1E.

The UV absorption spectra of *Spartium junceum* L. dye solutions after 1 cycle of extraction (1E) are shown in Figure 3. It shows 4 bands in the region of λ_max_ 224, 268, 308 and 346 nm which are ascribed to benzoyl and cinnamoyl bands characteristic for flavonoids [31]. Flavonoids are comprised of two benzene rings linked via a heterocyclic pyrane ring visible as a band at 224 nm which correlates well with the literature [12,32]. In the paper published by Amat, A. et al., the UV absorbance band of luteolin was measured in acidic media and found to be 348 nm which is in line with our measurements [33]. The peak at 268 nm is characteristic of flavonols, the subclass of flavonoids whose representative is quercetin [34].

**Figure 4:**
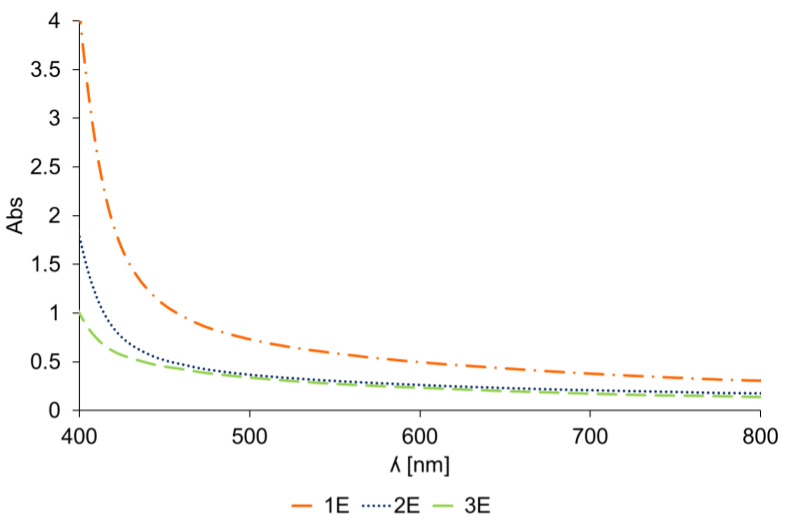
The VIS absorption spectra of 1st extraction (1E), 2nd extraction (2E) and 3rd extraction (3E).

The absorbance spectra of *Spartium junceum* L. dye solutions after 3 cycles of extraction are shown in Figure 4. As can be seen, the dye solution from the first extraction shows the highest absorption intensity at 400 nm wavelength in the visible area, which proved the presence of the highest amount of extracted pigments. According to the literature [16], *Spartium junceum* L. flowers contain luteolin and quercetin dyes responsible for its yellow color. Flavonoids like luteolin and quercetin are expected to have the highest absorbance in the yellow area (from 390 to 450 nm) [12].

#### 3.2.4. FT-IR Spectra of Crude Dye

The color of SJL flowers is directly connected to the presence of flavonoids, which are mainly present in yellow colored parts of plants [15,16]. FTIR technique was applied for possible differentiation of flavonoids subclasses presented in examined crude dye [12,28].

**Figure 5:**
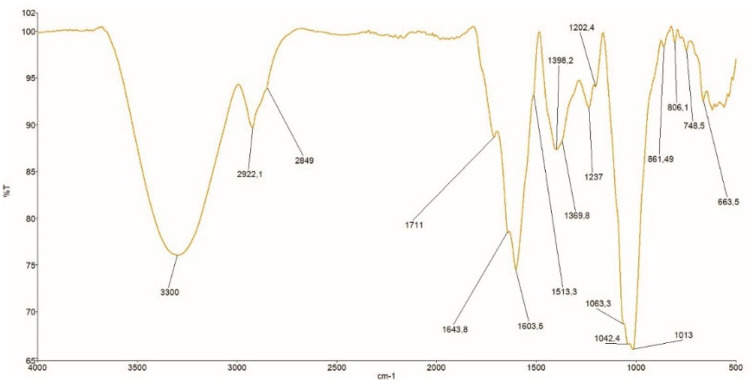
The transmission FTIR spectra of the first extraction (1E) crude dye.

FTIR spectra of crude dye after the first extraction are presented in Figure 5.

This spectrum can be divided into six regions:

1. 4000–2500: associated with the stretching vibrations of OH functional group of phenols (3300 cm^−1^) and aromatic CH and CH_2_ stretching vibrations of alkenes (2849 and 2922 cm^−1^) [35];
2. 2499–1800: this region didn’t show any applicable spectral information;
3. 1799–1630: assigned to double bond stretching vibrations (band at 1711 cm^−1^ corresponding to C = O stretch of ketone, and band at 1643 cm^−1^ corresponding to carbonyl group characteristic for flavones, i.e., for luteolin [36,37];
4. 1629–1400: assigned to C = C stretching of the aromatic ring (1603 cm^−1^ and 1513 cm^−1^);
5. 1399−1200: associated with C–OH deformations vibrations of phenols (1398 cm^−1^ and 1369 cm^−1^) and C–O stretching vibrations of aromatic ethers in phenols (1237 cm^−1^) and C–O stretching band due to pyranose structure (1202 cm^−1^) [38];
6. 1199–700: assigned to C–O stretching vibrations (1063 and 1042 cm^−1)^, C−OH stretching vibrations characteristic for flavanols, i.e., for quercetin (1013 cm^−1)^ and out of plane CH deformation vibrations of the functional groups mainly from carbohydrates (region from 900 to 700 cm^−1)^ [12,28,39,40].

The spectral area from 1799 and 700 cm^−1^ is called the ‘’fingerprint region’’ and is notable for the large number of infrared bands and is very important to characterize the sample’s chemical structure. 

### 3.3. Analysis of Fabrics after Pre-Mordanting, Dyeing and Washing 

#### 3.3.1. Color Measurement

**Figure 6:**
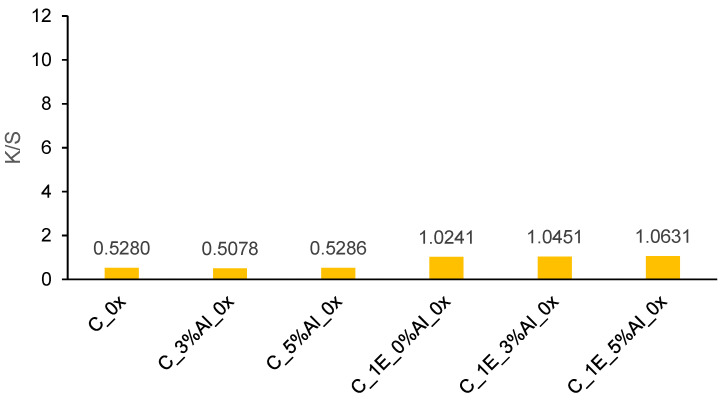
K/S values of cotton samples which are untreated (C_0x), treated only with mordants (C_3%Al_0x and C_5%Al_0x) or dyed with (C_1E_3%Al_0x and C_1E_5%Al_0x) and without mordants (C_1E_0%Al_0x).

**Figure 7:**
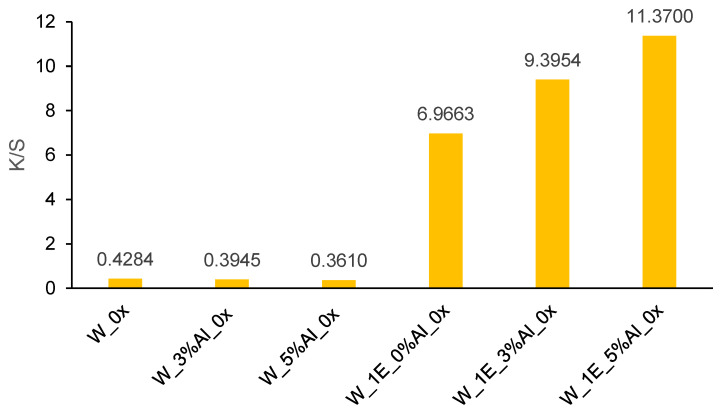
K/S values of wool samples which are untreated (W_0x), treated only with mordants (W_3%Al_0x and W_5%Al_0x) or dyed with (W_1E_3%Al_0x and W_1E_5%Al_0x) and without mordants (W_1E_0%Al_0x).

K/S values of cotton and wool fabrics are presented in Figure 6 and Figure 7. Figure 6 shows the effect of different mordant concentrations on dyed and undyed cotton fabrics. It is also visible that different aluminum mordant concentration does not significantly affect the K/S value. Vankar, P.S. et al. [31] stressed out that alkaline pH allows the strongest affinity of Al^3+^ metal to natural dye than acidic pH. Consequently, such low color strength is due to poor metal bonding with cellulose substrate and natural dye. Furthermore, Glogar, M.I. et al. presented in their paper [41] how the sample surface affects color strength as well. Smooth fabrics like cotton produce lower K/S compared to fabrics with a rougher surface (e.g., wool).

Figure 7 shows the effect of different mordant concentrations on dyed and undyed wool fabrics. The undyed wool substrate shows slightly yellow color. It was observed that by increasing the alum concentration up to 3% and 5% on undyed wool substrates, the color strength was decreased by 7.91% and 15.73% indicating poor bonding between wool and metal ions. Metal ions from alum mordant cause linking between dye and wool substrate by forming covalent bonding between functional groups of the dye molecule (OH, C = O) and protein fiber (NH_2_, COOH). Dyed wool fabrics treated with 3% and 5% alum mordant show an increase in K/S value compared to dyed wool fabric without mordant for 34.87% and 63.21%, respectively.

A huge difference between cotton and wool fabrics regarding the color strength is related to the charge of the dye and fabric. In this research, the acidic dye was extracted from the *Spartium junceum* L. flowers which has an anionic character and negative charge. Cotton fabric has a negative charge as well. Since the same charges repel each other, it was apparent that cotton fabric with current conditions would not show sufficient color strength compared to the wool fabric which is positively charged and therefore much more prone to fiber/mordant/dye crosslinking [42,43,44,45].

Table 3 presents that pre-mordant and undyed cotton samples show a minor decrease in lightness, chroma and hue for 0.19%, 11.47% and 1.19%, respectively for sample pre-mordant with 3% alum.

They also show a decrease in lightness, chroma and hue for 0.46%, 12.67% and 1.14%, respectively for sample pre-mordant with 5% alum, both regarding the reference samples C_0x. Subjective visual assessment of dyed cotton samples matches the objective measurement and gives light yellow color. These samples show no significant differences in hue (h° is in the range from 95.49 to 95.86) and lightness values (L* is in the range from 90.01 to 90.09). Dyed cotton sample without previous mordanting shows the highest lightness value (L* = 90.09) and the lowest chroma value (C* = 17.60) within series of dyed samples. The increase in chroma value is noticeable for dyed cotton samples, which are pre-mordant with 3% and 5% alum for 16.76% and 21.53%, respectively, regarding the dyed cotton sample without previous mordanting (C_1E_0%Al_0x). According to the paper published by Repon, M.R. et al. pre-mordanting of cotton fabric with 5% alum shows slightly positive impact on color saturation of dyed cotton fabric but negative impact on the environment since Al^3+^ metal from alum mordant shows lower exhaustion on material surface due to its lower coordination number [11].

Table 4 presents that pre-mordant and undyed wool samples show a minor decrease in lightness and chroma for 0.36% and 7.85%, respectively, while a minor increase in hue for 1.17%, for sample pre-mordant with 3% alum regarding the reference sample W_0x occurs.

Wool sample pre-mordant with 5% alum shows an increase in lightness and hue for 0.87% and 1.20%, respectively and a decrease in chroma for 8.78%, regarding the reference sample W_0x. Dyed wool samples show higher intensity in coloration than cotton samples which is assessed by subjective visual method and confirmed objectively by color coordinate measurements. Comparing cotton and wool dyed samples noticeable decrease in lightness (L* was approx. 90 for cotton and 70 for wool samples) and hue (h° was approx. 95 for cotton and 82 for wool samples) occurs while at the same time increase in chroma values (C* was approx. 20 for cotton and 46 for wool samples) happens. Haji, A. et al. have shown in their research paper mechanism of attachment of quercetin to wool structure assisted with the addition of alum mordant. One molecule of mordant binds the protein fiber while at the same time holding one or two molecules of dye with it, causing an increase of the affinity between wool fiber and dye and its color yield as well [46].

Similar to cotton, dyed wool sample without previous mordanting shows the highest lightness value (L* = 71.70) and the lowest chroma value (C* = 38.74) within series of dyed samples. Samples which are dyed and pre-mordant with 5% alum show lowest lightness value (L* = 69.96) and highest chroma (C* = 51.64).

The impact of multiple washing cycles on the color fastness of cotton and wool dyed fabrics was monitored through an objective color measurement technique. Colorfastness to washing is an important factor since most dyed fabrics are frequently washed after their usage. Results were presented in Table 5, Table 6, Table 7, Table 8, Table 9, Table 10, Table 11, Table 12, Table 13, Table 14, Table 15, Table 16, Table 17 and Table 18.

The difference in the sensation of color after washing was presented by Delta E* values. Table 5 shows there are no significant differences among washed and unwashed samples since Delta E* is lower than 2 which is an indicator that the differences are within the tolerance limits. 

Table 6 and Table 7 show that metal (Al) from alum mordant binds well and firmly to the cotton material, especially when applied in higher concentrations (5%). Comparing only the best results from Table 6 and Table 7 (samples after 1 washing cycle) with an untreated cotton sample from Table 5 (Delta E* = 0.55) it can be seen that Delta E* has increased and amounts 1.33 and 1.70, respectively.

Table 8 shows high colorfastness to washing (after 1st washing cycle) of cotton dyed samples without pre-mordant (Delta E* = 20.15). Delta E* higher than 2 indicates that differences in coloration are visible with the naked eye and are outside the tolerance limits. A higher number of washing cycles influences Delta E* values indicating a greater color change of sample after 5 washing cycles (Delta E* = 25.52).

Results of pre-mordant and dyed cotton material are presented in Table 9 and Table 10. It is noticeable that metal (Al^3+^) from alum mordant does not make strong chemical bonds with dye since Delta E* values increase regarding the higher concentration of alum and higher number of washing cycles, as well. This is also confirmed with differences in lightness and hue values, which directly affect Delta E*. The difference in lightness value (dL*) has the smallest influence on the total difference in coloration (dE*). The dL* values are under 5.00 while differences in hue (dH*) are increasing with a higher number of washing cycles and are in the range of approx. 16.00 to 20.00 indicating a noticeable change in samples’ color.

Visual assessment of colorfastness to washing can be seen in Table 11. In the first part of Table 11, undyed cotton samples were presented and the effect of washing on its discoloration is almost negligible.

Dyed cotton samples were presented in the second part of Table 11. Discoloration of samples after the washing process is significant especially since the color change is quite noticeable after 5 washing cycles. 

Table 12 shows that there are significant differences among washed samples after 3 and 5 washing cycles and unwashed samples. Delta E* is lower than 2 for wool untreated samples after 1st washing cycle (Delta E* = 1.24) which is an indicator that the differences are within the tolerance limits.

Table 13 and Table 14 show that metal (Al) from alum mordant binds well and firmly to the wool material, especially when applied in higher concentrations (5%). Comparing only the best results from Table 13 and Table 14 (samples after 1 washing cycle) with the untreated wool sample from Table 12 (Delta E* = 1.24) it can be seen that Delta E* has decreased by 100% and amounts 0.78 and 0.74, respectively.

Table 15 shows better colorfastness to washing of wool dyed samples without pre-mordant (Delta E* = 2.39) in comparison to the cotton sample treated under the same conditions. Delta E* higher than 2 indicates that differences in coloration are visible with the bare eye and are outside the tolerance limits.

Results of pre-mordant and dyed wool material are presented in Table 16 and Table 17. It is interesting that wool samples treated with mordant show a decrease in Delta E* values regarding a higher number of washing cycles.

The best result is shown in Table 16 by the sample treated with 3% alum after 5 washing cycles (Delta E* = 0.87). These results confirm that metal (Al) from alum mordant make strong chemical bonds with wool substrate and dye since Delta E* values decrease in comparison to Delta E* values of the cotton samples treated in the same way. These results correlate well with the research of Haji, A. [47] and Pour, R.A. [48] who have shown that optimization of metal mordants concentrations and usage of lower concentration can improve colorfastness to washing. Although cotton samples did not show significant differences among various mordant concentrations, wool samples show that the concentration of mordant has a significant effect on the obtained results and on the dyeing effects as well as colorfastness properties. Lightness values are dependent on both mordant concentration and number of washing cycles [49]. Higher mordant concentration influences lower lightness value while a higher number of washing cycles shows a slightly higher lightness value. Considering only wool samples pre-mordant with 3% alum and dyed, its L* value is in the range from 70.91 for unwashed samples to 71.56 for samples after 5 washing cycles (Table 4). All the dyed samples show color coordinates in a red-yellow zone. The a* is increasing, while b* is decreasing with higher numbers of washing cycles, except for the sample treated with 5% alum where is visible a minor increase in b* value—from 51.02 for the unwashed sample to 54.19 for sample after 5 washing cycles. Hue angle shows the highest values for unwashed samples and for the sample treated with 3% alum.

Visual assessment of colorfastness to washing for wool fabrics was performed as well and presented in Table 18. The effect of the washing process on its color change is visible within L* value which shows lighter shades with regard to higher mordant concentration (L* from 90.45 for unmordant to 91.24 for the sample treated with 5% alum) and darker shades with regard to a higher number of washing cycles (L* from 90.45 for unwashed wool without a mordant sample to 88.86 for the same sample after 5 washing cycles).

Discoloration of woolen samples after the washing process is significantly lower than was the case with cotton samples. According to Li, Y.V. et al. if the natural dye is bonded with mordant forming a stable dye-metal complex then the water solubility of the dye is lower and the color is less likely to bleed out on washing process [50]. Lightness value was decreasing while increasing mordant concentration (L* from 71.70 for unmordant sample to 69.96 for the sample treated with 5% alum) providing darker shades of wool fabrics which are in contrast to increasing the L* value when increasing the number of washing cycles (L* from 70.91 for unwashed and with 3% alum pre-mordanted sample to 71.56 for the same sample after 5 washing cycles) providing a little bit lighter shades. 

#### 3.3.2. FT-IR Spectra of Dye Wool Fabrics

**Figure 8:**
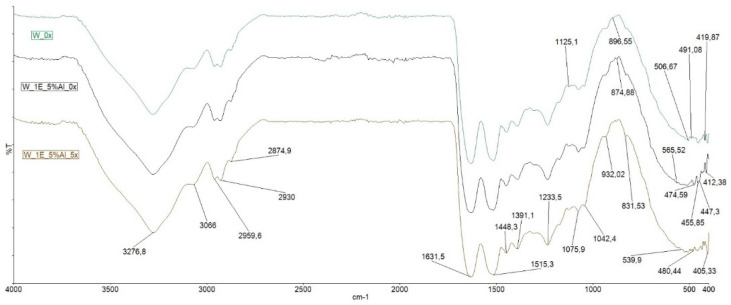
FTIR spectra of wool fabrics, where W_0x is reference fabric, W_1E_5%Al_0x is pre-mordant with 5% alum, dyed and unwashed wool fabric and W_1E_5%Al_5x is the same sample as previous but after 5 washing cycles.

FTIR spectra of reference (unmordant, undyed and unwashed) and dyed wool fabrics are presented in Figure 8. Spectra of all samples show absorption peaks at 3276 cm^−1^ assigned to the NH stretching vibrations, 1631 cm^−1^ assigned to amide-I (C = O stretching), 1515 cm^−1^ assigned to amide-II (NH bending and CN stretching) and 1075 cm^−1^ assigned to CN stretching and NHG bending of amide-III [51]. The peak at 1391 cm^−1^ is assigned to the amino acid (COO-) functional group. A peak at 1233 cm^−1^ is assigned to CH_2_ and CH_3_ bending. Dyed samples showed a little bit lower intensity of peaks characteristic for amide-I and amide-II compared to untreated wool indicating the involvement of amine functional groups within the wool, alum and dye molecules [45]. Untreated wool shows a peak at 1515 cm^−1^ (peak characteristic for amide-II) which closely corresponds to the peak at 1513 cm^−1^ of investigated dye solution. An increase of intensity of a very weak band at 1125 cm^−1^ which is assigned to amide-II due to CN stretch is visible in the pre-mordant and dyed wool spectra indicating the effect of alum mordant. Peak visible in an untreated wool sample at 506 cm^−1^ is assigned to the S-S bond. In pre-mordant and dyed samples this peak hasn’t been detected since the area from 500–600 cm^−1^ showed several peaks of minor intensity characteristic for sulfate absorption and Al-O stretching vibration from alum mordant (539 and 565 cm^−1^) [48,52,53].

## 4. Conclusions

The paper proved that the extracted dye from *Spartium junceum* L. is an acidic dye (mordant dye) that is successfully used for the treatment of protein fibers. Cellulose fibers can also be dyed with acidic dye, but optimization of conditions is required. Dyed wool samples show higher intensity in coloration than cotton samples. Wool samples that are dyed and pre-mordant with 5% alum show the lowest lightness value (L* = 69.96) and highest chroma (C* = 51.64) regarding the other dyed samples. Discoloration of wool samples after the washing process is significantly lower than was the case with cotton samples. The best results were achieved with wool samples treated with 3% alum where Delta E* after 5 washing cycles was 0.87 and within the tolerance limits. A further conclusion is in the possibility to reduce the concentration of mordant to 3% to obtain satisfactory results regarding colorfastness. Otherwise, approx. 15% of mordant is added, and this significant reduction favors potentially increased negative environmental impact. This research shows wide practical application due to the aspects of circular economy, textile care and environmental friendliness. According to the zero waste model, this research is focused on the total utilization of the SJL plant. The majority of the SJL plant usage has been successfully processed in our previous research. The least used part of the SJL plant is found out to be a valuable source of raw material for natural dye production. Such dyes show better affection to protein fibers and consequently better color fastness, which enables its application for dyeing the textile materials suitable for multi-cycle washing. Positive results gained after reduction of alum mordant concentration influence usage of SJL natural dye in a more environmentally friendly manner and allow its response to the stringent requests of various EU regulations. Nonetheless, future research will go in the direction of replacing a synthetic mordant with a more environmentally friendly mordant. Furthermore, these findings lead us to the interdisciplinary approach and wider research on the usage of energy crop cultures which are investigated within the BIOCOMPOSITES project, such as *Miscanthus x giganteus*, *Sida hermaphrodita* or *Arundo donax,* as a valuable resource for dyes extracted from their leaves, while at the same time facilitates their eventual application in practice.

## Figures and Tables

**Table 1:** Technical characteristic parameters of used fabrics.

Label	Composition	Producer	Weave	Density (cm^−1^)		Weight (g/m^2^)
				Warp	Weft	
C	100% Cotton	Čateks d.o.o., Čakovec, Croatia	Plain	26	25	191.45
W	100% Wool	Tekstilpromet d.d., Zagreb, Croatia	Plain	22	22	118.16

**Table 2:** Crude dye yield in the investigated dye solutions.

Dye Solutions	Mass of the Crude Dye (g)	Crude Dye Yield (%)
1E	52.8	4.8
2E	25.98	2.4
3E	12.48	1.1

**Table 3:** Color coordinates of cotton samples dyed with SJL aqueous extract with and without mordants before washing.

Samples	L*	a*	b*	C*	h
C_0x	97.27	3.33	−11.20	11.68	286.54
C_3%Al_0x	97.09	2.35	−10.07	10.34	283.14
C_5%Al_0x	96.82	2.34	−9.93	10.20	283.27
C_1E_0%Al_0x	90.09	−1.73	17.52	17.60	95.65
C_1E_3%Al_0x	90.02	−2.10	20.45	20.55	95.86
C_1E_5%Al_0x	90.01	−2.05	21.30	21.39	95.49

**Table 4:** Color coordinates of wool samples dyed with SJL aqueous extract with and without mordants before washing.

Samples	L*	a*	b*	C*	h
W_0x	90.45	−1.04	12.83	12.87	94.62
W_3%Al_0x	90.12	−1.18	11.80	11.86	95.73
W_5%Al_0x	91.24	−1.18	11.68	11.74	95.76
W_1E_0%Al_0x	71.70	5.01	38.42	38.74	82.56
W_1E_3%Al_0x	70.91	6.54	47.31	47.76	82.13
W_1E_5%Al_0x	69.96	7.98	51.02	51.64	81.11

**Table 5:** Change in color coordinates of the cotton sample without mordants after 1st, 3rd and 5th washing cycle (compared with C_0x).

Samples	dL*	dC*	dH*	dE*
C_1x	−0.17	−0.46	0.25	0.55
C_3x	−0.36	−0.78	0.33	0.92
C_5x	−0.29	−0.23	0.19	0.42

**Table 6:** Change in color coordinates of the cotton sample treated with 3% Al mordants after 1st, 3rd and 5th washing cycle (compared with C_3%Al_0x).

Samples	dL*	dC*	dH*	dE*
C_3%Al_1x	−0.03	1.06	0.80	1.33
C_3%Al_3x	0.09	1.19	0.75	1.41
C_3%Al_5x	0.13	1.48	0.69	1.64

**Table 7:** Change in color coordinates of the cotton sample treated with 5% Al mordants after 1st, 3rd and 5th washing cycle (compared with C_5%Al_0x).

Samples	dL*	dC*	dH*	dE*
C_5%Al_1x	0.24	1.51	0.74	1.70
C_5%Al_3x	0.32	1.55	0.79	1.77
C_5%Al_5x	0.40	1.67	0.71	1.86

**Table 8:** Change in color coordinates of cotton samples dyed with SJL aqueous extract without mordants after 1st, 3rd and 5th washing cycle (compared with C_1E_0%Al_0x).

Samples	dL*	dC*	dH*	dE*
C_1E_0%Al_1x	3.78	−18.36	−7.41	20.15
C_1E_0%Al_3x	4.64	−15.08	−18.69	24.46
C_1E_0%Al_5x	5.12	−14.15	−20.61	25.52

**Table 9:** Change in color coordinates of cotton samples dyed with SJL aqueous extract with 3% Al mordants after 1st, 3rd and 5th washing cycle (compared with C_1E_3%Al_0x).

Samples	dL*	dC*	dH*	dE*
C_1E_3%Al_1x	3.45	−18.87	−5.39	19.93
C_1E_3%Al_3x	4.32	−17.13	−16.09	23.90
C_1E_3%Al_5x	5.00	−15.61	−19.79	25.70

**Table 10:** Change in color coordinates of cotton samples dyed with SJL aqueous extract with 5% Al mordants after 1st, 3rd and 5th washing cycle (compared with C_1E_5%Al_0x).

Samples	dL*	dC*	dH*	dE*
C_1E_5%Al_1x	3.37	−19.38	−4.38	20.15
C_1E_5%Al_3x	4.42	−17.90	−16.68	24.87
C_1E_5%Al_5x	4.84	−17.07	−18.78	25.84

**Table 11:** Visual assessment of colorfastness to washing.

**Washing**	**Cotton without Mordant**	**Cotton 3% Mordant**	**Cotton 5% Mordant**
0x	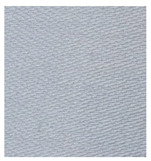	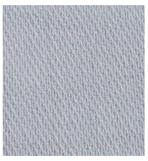	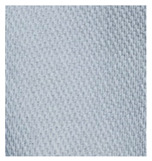
5x	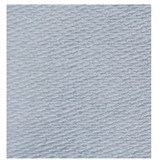	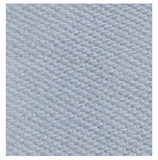	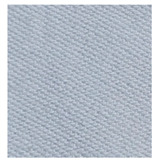
**Washing**	**Dyed Cotton without Mordant**	**Dyed Cotton 3% Mordant**	**Dyed Cotton 5% Mordant**
0x	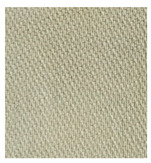	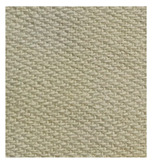	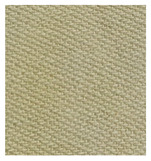
5x	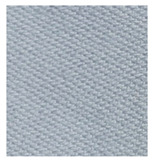	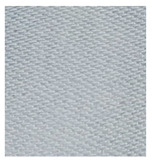	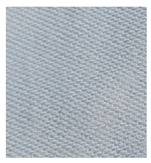

**Table 12:** Change in color coordinates of the wool sample without mordants after 1st, 3rd and 5th washing cycle (compared with W_0x).

Samples	dL*	dC*	dH*	dE*
W_1x	−0.96	0.79	−0.03	1.24
W_3x	−1.51	2.02	−0.38	2.55
W_5x	−1.59	2.50	−0.48	3.00

**Table 13:** Change in color coordinates of wool sample treated with 3% Al mordants after 1st, 3rd and 5th washing cycle (compared with W_3%Al_0x).

Samples	dL*	dC*	dH*	dE*
W_3%Al_1x	0.22	0.75	0.01	0.78
W_3%Al_3x	−1.09	2.45	−0.40	2.71
W_3%Al_5x	−1.64	3.58	−0.80	4.02

**Table 14:** Change in color coordinates of wool sample treated with 5% Al mordants after 1st, 3rd and 5th washing cycle (compared with W_5%Al_0x).

Samples	dL*	dC*	dH*	dE*
W_5%Al_1x	−0.46	0.58	0.08	0.74
W_5%Al_3x	−1.47	1.69	−0.04	2.24
W_5%Al_5x	−1.84	2.60	−0.23	3.19

**Table 15:** Change in color coordinates of wool sample dyed with SJL aqueous extract without mordants after 1st, 3rd and 5th washing cycle (compared with W_1E_0%Al_0x).

Samples	dL*	dC*	dH*	dE*
W_1E_0%Al_1x	−1.03	1.60	−1.46	2.39
W_1E_0%Al_3x	−0.35	−0.90	−1.52	1.80
W_1E_0%Al_5x	0.56	−3.22	−1.55	3.61

**Table 16:** Change in color coordinates of cotton samples dyed with SJL aqueous extract with 3% Al mordants after 1st, 3rd and 5th washing cycle (compared with W_1E_3%Al_0x).

Samples	dL*	dC*	dH*	dE*
W_1E_3%Al_1x	−1.58	7.14	−1.31	7.43
W_1E_3%Al_3x	−0.62	4.71	−0.98	4.85
W_1E_3%Al_5x	0.65	−0.27	−0.51	0.87

**Table 17:** Change in color coordinates of wool sample dyed with SJL aqueous extract with 5% Al mordants after 1st, 3rd and 5th washing cycle (compared with W_1E_5%Al_0x).

Samples	dL*	dC*	dH*	dE*
W_1E_5%Al_1x	−2.13	8.68	−1.37	9.04
W_1E_5%Al_3x	−1.28	8.80	−1.01	8.94
W_1E_5%Al_5x	−0.08	3.25	−0.29	3.27

**Table 18:** Visual assessment of colorfastness to washing.

**Washing**	**Wool without Mordant**	**Wool 3% Mordant**	**Wool 5% Mordant**
0x	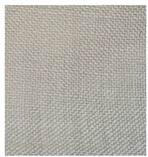	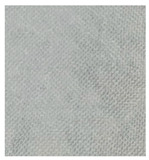	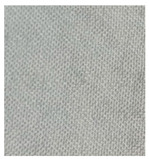
5x	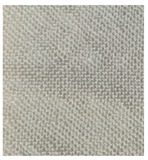	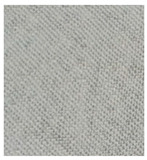	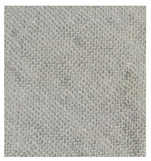
**Washing**	**Dyed Wool without Mordant**	**Dyed Wool 3% Mordant**	**Dyed Wool 5% Mordant**
0x	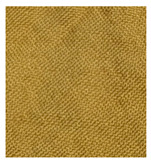	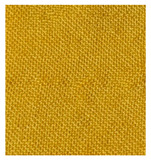	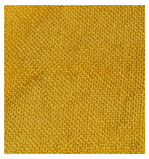
5x	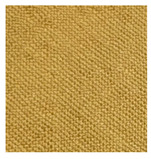	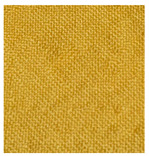	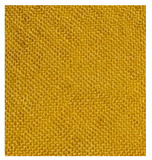

## Data Availability

Data available in a publicly accessible repository.
